# Electrodialysis with Bipolar Membranes for the Generation of NaOH and HCl Solutions from Brines: An Inter-Laboratory Evaluation of Thin and Ultrathin Non-Woven Cloth-Based Ion-Exchange Membranes

**DOI:** 10.3390/membranes12121204

**Published:** 2022-11-29

**Authors:** Tamara León, Syed Abdullah Shah, Julio López, Andrea Culcasi, Lluis Jofre, Andrea Cipollina, José Luis Cortina, Alessandro Tamburini, Giorgio Micale

**Affiliations:** 1 Chemical Engineering Department, Escola d’Enginyeria de Barcelona Est (EEBE), Universitat Politècnica de Catalunya (UPC)-BarcelonaTECH, C/Eduard Maristany 10-14, Campus Diagonal-Besòs, 08930 Barcelona, Spain; 2 Barcelona Research Center for Multiscale Science and Engineering, Campus Diagonal-Besòs, 08930 Barcelona, Spain; 3 Dipartimento di Ingegneria, Università degli Studi di Palermo, Viale delle Scienze Ed. 6, 90128 Palermo, Italy; 4 Department of Fluid Mechanics, Escola d’Enginyeria de Barcelona Est (EEBE), Universitat Politècnica de Catalunya (UPC)-BarcelonaTECH, C/Eduard Maristany 10-14, Campus Diagonal-Besòs, 08930 Barcelona, Spain

**Keywords:** BMED, acid-base production, brine management, brine intensification, ion-exchange membrane, electro-membrane

## Abstract

The SEArcularMINE project aims to recover critical raw materials (CRMs) from brines from saltworks, thus facing a CRM shortage within Europe. To promote a fully circular scheme, the project valorises concentrated brines using electrodialysis with bipolar membranes (EDBM) to generate the required amounts of reactants (i.e., acids and bases). Regarding the performances of new non-woven cloth ion-exchange membranes (Suez): (i) an ultra-thin non-woven polyester cloth and (ii) a thin polypropylene cloth acting as the support structures were assessed. Additionally, the anion layer includes a catalyst to promote the water dissociation reaction. The effect of current density (100, 200, and 300 A m^−2^) on the performance of two combinations of membranes in an inter-laboratory exercise using 2 M NaCl was evaluated. According to statistical analysis ANOVA, there was an agreement on the results obtained in both laboratories. NaOH/HCl solutions up to 0.8 M were generated working at 300 A m^−2^ using both combinations of membranes. Regarding the performance parameters, stack set-ups incorporating thin polypropylene membranes showed lower specific energy consumption (SEC) and higher specific productivity (SP) than ultra-thin polypropylene ones. Hence, for ultra-thin polypropylene membranes, SEC was reported to be between 2.18 and 1.69 kWh kg^−1^_NaOH_ and SP between 974 and 314 kg m^−2^ y^−1^.

## 1. Introduction

In recent years, the world population has continuously grown, posing new challenges for sustainable development. According to the European Union (EU) Circular Economy action plan, the raw material consumption is expected to double by 2060, while waste production is expected to increase by 70% by 2050 [1]. Furthermore, the European Union has classified some raw materials as “critical” due to (i) their high economic importance (EI) and (ii) high supply risk (SR) [1]. The critical raw materials (CRMs) list [2] aims to evaluate the material criticality of a number of materials according to the European Commission’s criticality methodology. According to the Green Deal action plan, circular economy strategies should be applied, thus allowing [2] to reduce the reliance on non-European countries, as over 90% of raw materials are imported [3].

Seawater mining has been proposed as one of the most promising alternative ways to recover CRMs (e.g., magnesium, lithium) from seawater [4]. For example, brines from the desalination industry can be used effectively for materials recovery because of the high concentration of ions in the solution. These saline wastewaters should be appropriately treated using zero liquid discharge (ZLD) or minimum liquid discharge (MLD) methods to meet EU emissions targets. The ZLD and MLD approaches rely on pre-treatment and pre-concentration steps to achieve 95% water recovery. However, a ZLD strategy also includes post-concentration processes based on energy-intensive practices to achieve zero liquid waste [5]. Nevertheless, only in a few previous works have ZLD and MLD strategies focused on valorising these concentrated streams to recover or produce valuable compounds (e.g., recover single [6] or mixed salts [7]). Consequently, the need to develop the on-site chemical production of commodities, such as strong acids and bases, has identified the use of electrodialysis with bipolar membranes (EDBM) as a technological driver to achieve this objective through the valorisation of waste brines. 

**Figure 1:**
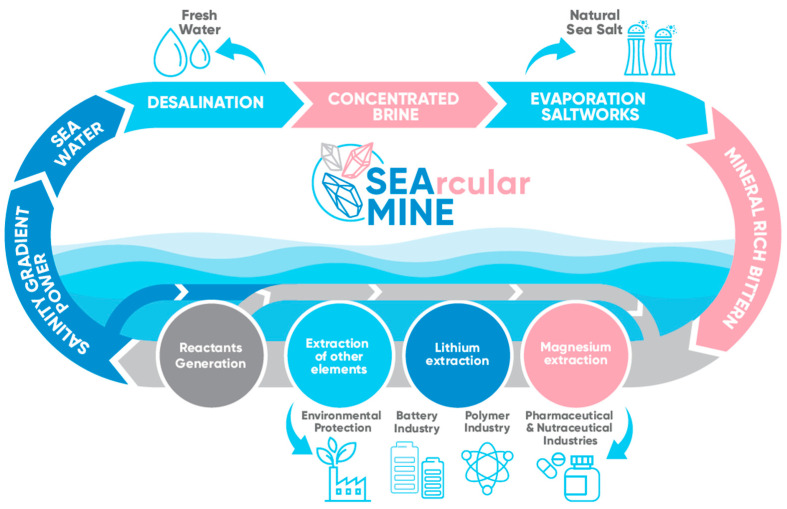
SEArcularMINE concept scheme (from [9]).

Although EDBM is an electro-membrane technology postulated for decades to produce acids and bases from brines [8], two main technical limitations slow down its application at full-scale, such as (i) the lack of high-performance ion-exchange membranes (IEMs) (e.g., cationic (CEM), anionic (AEM), and bipolar (BPM)), and (ii) the economic feasibility of the produced chemicals. It should be mentioned that the market cost of the strong acids, such as HCl and H_2_SO_4_, and strong bases, such as NaOH, are affected by the transportation cost. The need to mature this technology is supported by developing: (i) a deeper evaluation of the IEMs properties; (ii) new processing routes of brines as potential feed streams to be valorised; and (iii) new concepts of material circularity and sustainability to overcome the economic feasibility barriers. As an example, the efforts by the EU in promoting circular approaches, the recovery of raw materials, and/or the production of chemicals in the case of the project creating a sustainable circular approach to mineral extraction, SEArcularMINE (Figure 1) [9].

Specifically, the SEArcularMINE project aims to use an innovative circular approach to valorise bitterns from saltworks to extract CRMs, such as magnesium [10], lithium [11,12], and other elements [13]. As the SEArcularMINE project is based on a fully circular concept, the reactants needed for their processes must be produced in situ. Therefore, to avoid the need for external chemical reagents, the concentrated and exhausted brines will be used in the EDBM process to generate the necessary alkaline and acidic reactants [14].

**Figure 2:**
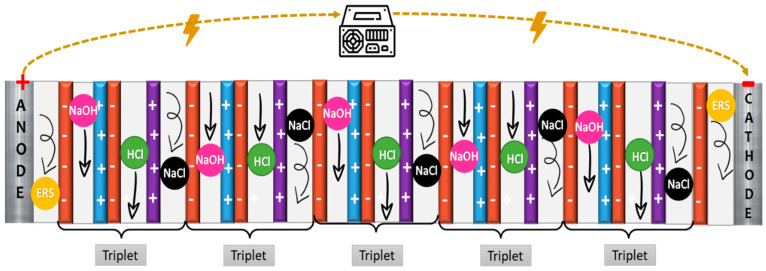
EDBM-unit scheme containing 5 triplets.

An EDBM unit is composed of a stack of repeating units, namely triplets, which are made up of three channels (i.e., acid, alkaline, and salt), a monopolar CEM, a monopolar AEM, and a BPM as illustrated in Figure 2. During operation, when a direct electric current is applied, the water molecules in the junction of the anion and cation-exchange layers of the BPM are dissociated into protons (H^+^) and hydroxide (OH^-^) ions. The cations and anions in the feed saline solution migrate through the CEM and AEM towards the cathode and anode, respectively, ultimately producing acidic and alkaline solutions [15].

EDBM has received significant attention from many researchers due to its wide range of applications [16]. The production of inorganic acids and bases from salts [17,18] is one of the most common (semi)-industrial applications of EDBM [17]. EDBM has proven to be a promising and efficient method for chemical production due to its simplicity, as it requires just two inputs: electric energy and a saline feed [18]. As an example, several authors have evaluated the performance of EDBM for the valorisation of brines [19,20,21,22]. Yang et al. [19] used seawater reverse osmosis (SWRO) brines to produce acid and base solutions at the laboratory scale, using CEMs and AEMs provided by Qianqiu^®^ and BPMs provided by Fumatech^®^. In order to avoid scaling, the authors pre-treated the SWRO brine with NaOH/CO_2_(g) to remove Mg and Ca (<1.2 mg/L). Regarding EDBM tests, they achieved a concentration of acid and base solutions higher than 1 M with an average Specific Energy Consumption (SEC) of 7 kWh kg^−1^_HCl_ and Current Efficiencies (CEs) in the range of 50–60%. In a similar work, Reig et al. [20] treated a SWRO brine in an electrodialysis unit, obtaining a NaCl-concentrated solution free of divalent ions, which was fed to the EDBM. Tests were carried out in a lab-scale EDBM unit comprising PC Cell^®^ membranes to produce 2 M of HCl and NaOH working at current densities of 300–400 A m^−2^ and with SEC values of 1.8–3.6 kWh kg^−1^_NaOH_ and CEs of 60–80%. Herrero et al. [21] utilised a lab-scale EDBM unit equipped with RALEX^®^ monopolar membranes and Fumatech^®^ BPMs. In this study, they reported the production of acid and base solutions in the range of 3.3 mol L^−1^ to 3.6 mol L^−1^ from NaCl. These high concentrations were obtained by providing an electric current density of 1 kA/m^2^ working at a salt-to-acid (and base) volume ratio of 20, thus resulting in high SECs (20–40 kWh kg^−1^_HCl_). Finally, Davis et al. [22] investigated the use of Neosepta^®^ membranes, achieving a maximum concentration of less than 0.4 M of acid and a base with SECs in the range of 1–2 kWh kg^−1^_NaOH_ and 3.65 kWh kg^−1^_HCl_.

Nowadays, numerous BPMs are available from different manufacturers for EDBM applications; several research studies have analysed their performance and relevant properties [17,23,24]. As a result, the main challenges on EDBM are associated with the influence of the feed solution composition on membrane performance, particularly the presence of dissolved organic matter or scaling forming solutes in terms of operation problems (e.g., organic fouling, biofouling, and/or scaling). Other challenges were centred on modifying the membrane properties mainly by increasing the separation factors of monovalent to divalent ions [25,26], reducing the membrane electrical resistance [27], and decreasing the membrane costs by modifying the production processes. One option to improve the performances of IEM manufacturers (e.g., SUEZ) might be to use a non-woven polyester cloth as a substitute for a woven acrylic cloth. The non-woven cloth membranes have a lower electrical resistance that reduces the required voltage to achieve a target salinity reduction and, consequently, a lower energy consumption. Since the membranes are made of a non-woven cloth, they will not generate “strings” hanging on the exterior of the stack as the edge of the membranes become dry, as happens with woven cloths. A second option was to convert membranes that use woven cloths to a non-woven structure. AEM (AR103P) and CEM (CR61P) are cast on woven polypropylene cloth and are typically used in food and beverage processing applications. These membranes have been replaced by the AR103N and CR61N with a thin non-woven polypropylene cloth designed to provide a superior membrane product with lower power consumption and smoother surface to reduce scaling, fouling, and/or biofouling. Both membranes have been proposed as cation-selective and anion-selective layers of a BPM. The anion layer is treated with a catalyst to aid in the water dissociation function of the membrane [28].

Nevertheless, one of the problems associated with conventional (or standard) AEMs is the low acid concentration reached in the acid compartment (around 1 M) since protons are transported to the saline stream. In order to solve this, a new family of AEMs (AR118U) with proton-blocking properties has been developed, allowing for higher concentrations in the acid streams (e.g., up to 2 M).

This work presents a comprehensive comparison of two different membrane sets, namely Types A and B, for the first time. Specifically, Type A includes a family of IEMs incorporating thin non-woven polypropylene cloth (i.e., AR103N and CR61N), and Type B includes a family of IEM_S_ with ultra-thin non-woven polyester cloth (i.e., AR118U). The two sets of membranes were tested across varying operating conditions to assess the EDBM unit in terms of the key performance indicators that are of high importance in industrial-scale applications.

In both configurations, the BPM comprised a CEM and an AEM (a treated AR103N) on top of each other. The treated AEM is a proprietary commercial product, and the active layer is surface-treated with a catalyst, which may reduce the electrical consumption of the water dissociation reaction [29]. In this study, the EDBM was used to treat NaCl feed solutions mimicking bitterns generated by saltworks after Mg removal. The experiments were carried out in parallel using two lab-scale EDBM units with the same membrane stack and located at different laboratories (UNIPA and UPC) to assess the complete reproducibility and reliability of the results obtained. This work examined the influence of the applied current density and different membrane types on the SEC, CEs, and specific productivity (SP) related to NaOH. Finally, the results were compared to those of previous works that utilised the EDBM in similar applications and produced NaOH and HCl solutions.

## 2. Materials and Methods

### 2.1. Reagents and Membranes

NaCl (99.7% chemSolute), HCl (37% Merck), NaOH (98–100% Honeywell Flute), and Na_2_SO_4_ (99–100% Honeywell Flute) were used to prepare the synthetic solutions. All the reactants were of analytical grade.

The monopolar CEMs and the AEMs were kindly provided by SUEZ. The BPMs were assembled by combining a CEM (containing sulfonic acid groups) with an AEM (containing quaternary ammonium groups). In the surface-treated AEM, a catalyst enhances the water dissociation reaction. The needed voltage across the BPM for the water dissociation reaction is achieved by the action of an immobilised metal (e.g., iron II) acting as a catalyst [28]. Fine mesh polypropylene screen spacers with a thickness of 650 µm provided by SUEZ were adopted to give dimensional stability to the channels. The membranes selected for the experimental campaign of this work are the following (a) AEMs: AR118U and AR103N, (b) CEMs: CR61N, (c) BPMs: a combination of AR103N-treated and CR61N. Two different combinations of these membranes during stack assembly were tested: (i) Type A (AR118N, CR61N, AR103tr.) and (ii) Type B (AR103U, CR61N, AR103tr). Although all the membranes selected for this work are indeed non-woven cloths, there are differences among them. The membranes ended in U (i.e., AR118U) have ultra-thin non-woven polyester cloths as the support structures, while the others (ended in N) have thin polypropylene non-woven cloths acting as their support structures. N-type reports a slightly higher permselectivity; however, type U is thinner than type N. Table 1 contains additional information on each membrane’s characteristics and features of some commercially available membranes for comparison.

### 2.2. Laboratory-Scale Experimental Set-Up

Experiments were performed in two EDBM laboratory-scale units supplied by SUEZ (Electromat MkI ED STACK). Each EDBM unit incorporates a membrane stack (HDWR MK-1), with an active membrane area of 0.028 m^2^ (S-shape channel with 0.505 m length and 0.0555 m width). The membrane assembly contained the following components: (i) 5 repeating units, (ii) anode electrode (stainless steel with platinum coating), (iii) cathode electrode (stainless steel), and (iv) spacers (polypropylene).

The EDBM units are designed for a closed-loop (batch) configuration with four separate hydraulic circuits for the acid, base, salt, and ERS solutions. For that purpose, each hydraulic circuit was assembled with a volumetric pump and equipped with a pressure gauge. The EDBM process requires the application of an electric field as a driving force; so, each EDBM unit was also equipped with a power supply working under galvanostatic mode. During the experiments, several operating parameters were monitored: (i) temperature, (ii) external voltage and current, (iii) electrical conductivity of the electrolyte solutions, (iv) volume profiles over time for all the containers, (v) pressure, and (vi) volume flow rate. The target of the experiments was to reach target concentrations of NaOH of 0.4 M and 0.8 M.

**Figure 3:**
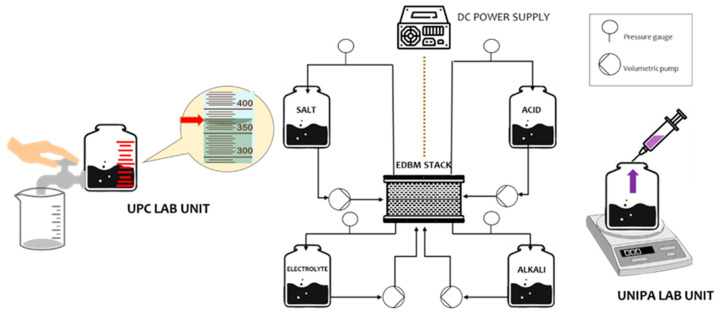
Scheme of EDBM units installed at UPC and UNIPA facilities along with a few procedural differences.

EDBM experiments were run in parallel at the same conditions at UNIPA and UPC premises with an almost identical set-up. However, there are some minor differences in each set-up, which are highlighted for completeness in Table 2 and displayed in Figure 3. It is worth noting that these differences did not affect the quality of the results or the reproducibility of the experiments.

### 2.3. Analytical Methodologies and Chemical Analysis

Sixteen samples on average (four per compartment, 5 mL each) were taken during each of the EDBM experiments. HCl and NaOH samples were analysed by acid/base titration. At UPC facilities, the titration was performed using an automatic titrator (T70, Mettler Toledo) equipped with an automated titration stand (Rondolino, Mettler Toledo); HCl and NaOH solutions 100 μM (previously standardised) were used as titrants. At UNIPA, titration was executed using as titrants a standard HCl 0.10 M and Na_2_CO_3_ 0.05 M solutions, and methyl orange as pH indicator.

The pH and conductivity evolution were monitored using conductivity meters (3320, Xylem Analytic Germany FmbH D-82362 Weilheim) and pH meters (3320, Xylem Analytic Germany FmbH D-82362 Weilheim; Crison pH Basic 20) at both premises.

### 2.4. Experimental Campaign

Initially, preliminary tests were performed to ensure that the experimental results were reproducible. These tests were performed under identical process conditions in terms of applied current, mean channel flow velocity, and initial acid, base, salt, and electrode rinse solution composition (see Table 3). Synthetic brine solutions (i.e., salt solutions) prepared with NaCl and distilled water were used in the EDBM experiments. The experiments were carried out with an initial non-zero concentration of the acid and base, as shown in Table 3, in order to reduce the initial electrical resistance of the electrolyte solutions. Then, the effect of varying (i) the combination of membranes (Types A and B) and (ii) the applied current density was investigated. Table 3 summarises the process conditions adopted in EDBM experiments.

### 2.5. EDBM Performance Parameters

All of the performance parameters refer to the amount of NaOH produced due to its economic and industrial interests.

Current efficiency (CE) is a fraction of the electric charges (introduced into the system) that are successfully converted into hydroxide ions [20]. *CE* was calculated as

(1) $$CE=\frac{\left(C_{b,NaOH,t}\cdot V_{b,t}-C_{b,NaOH,0}\cdot V_{b,0}\right)\cdot F}{I\cdot N\cdot t}\;,$$

where $C_{b,NaOH,t}\;$ (mol·L^−1^) is the concentration of NaOH at time *t*, $C_{b,NaOH,0}$ (mol·L^−1^) is the initial concentration of NaOH, $V_{b,t}$ (L) is the volume of the base solution at time *t*, $V_{b,0}$ (L) is the initial volume of the base solution, *F* (96,485 C·mol^−1^) is the Faraday constant, *I* (A) is the applied electric current to the stack, *N* is the number of triplets of the EDBM stack (i.e., 5 in this work) and *t* (s) is the processing time.

Specific energy consumption (*SEC*, kWh kg^−1^_NaOH_) refers to the amount of energy necessary to produce one kilogram of NaOH [8]. It was calculated according to

(2) $$SEC=\frac{I\cdot \int_{0}^{t}U\cdot dt}{3.6\cdot 10^{6}\cdot \left(C_{b,NaOH,t}\cdot V_{b,t}-C_{b,0}\cdot V_{b,0}\right)\cdot M_{NaOH}}=\frac{F\cdot \int_{0}^{t}U\cdot dt}{3.6\cdot 10^{6\;}\cdot M_{NaOH}\cdot CE\cdot N\cdot t}\;,$$

where *U* (V) is the external voltage and $M_{NaOH}$ (kg·mol^−1^) is the mole mass of NaOH.

Finally, it is important to relate the installed membrane area with the amount of NaOH that can be produced in a working year (assumed to be 8000 h) [8]. This relationship is critical when the process is scaled up to the industrial production level. This parameter is called specific productivity (*SP*, kg NaOH m^−2^ y^−1^) and was calculated according to

(3) $$SP=\frac{3.6\cdot 8\cdot 10^{6\;}M_{NaOH}\cdot \left(C_{b,NaOH,t}\cdot V_{b,t}-C_{b,NaOH,t_{0}}\cdot V_{b,t_{0}}\right)}{N\cdot 3\cdot S\cdot t}\;,$$

where *S* (m^2^) is the active membrane area.

The yield is the ratio between the produced amount of NaOH moles in the base compartment over the initial amount of NaCl moles in the salt compartment. It is given by,

(4) $$Yield=\frac{\left(C_{b,NaOH,t}\cdot V_{b,t}-C_{b,NaOH,t_{0}}\cdot V_{b,t_{0}}\right)}{C_{s,NaCl,t_{0}}\cdot V_{s,t_{0}}}\;,$$

The effective production represents the amount of produced NaOH per cubic metre of brine stream fed to the EDBM unit and was calculated by,

(5) $$Effective\;production=\frac{M_{NaOH}\cdot 1000\cdot \left(C_{b,NaOH,t}\cdot V_{b,t}-C_{b,NaOH,t_{0}}\cdot V_{b,t_{0}}\right)}{\;V_{s,t_{0}}}\;,$$

### 2.6. Reproducibility Analysis Procedure to Assess the Inter-Laboratory Results

The ANalysis of VAriance (ANOVA) was performed using single factor ANOVA utilizing an Excel^®^ spreadsheet to confirm reproducibility between UNIPA and UPC results. ANOVA permits the simultaneous comparison of more than two data groups to determine whether there is a correlation between them. Applying the ANOVA formula, the F-value is used to assess the variability between samples and within samples for multiple data groups [32]. Through the statistical analysis, the relevance of the factor of performing the EDBM experiments in different laboratories was analysed. Considering a significance level (α) of 0.05, results are considered statistically different if the F-value calculated (Fc) is less than the critical F-value (Fcr), which is called the null hypothesis [32].

## 3. Results

The results of the reproducibility analysis and the study of the effect of current density and different membrane types are presented and thoroughly discussed in this section.

### 3.1. Reproducibility Analysis

EDBM experiments were carried out in two different laboratories to demonstrate the reproducibility of the results and the reliability of the experimental method. Therefore, the information presented in this section is the result of the efforts of the two institutions. Additionally, the information displayed has been corroborated at least four times, thus demonstrating the accuracy of the experimental results.

**Figure 4:**
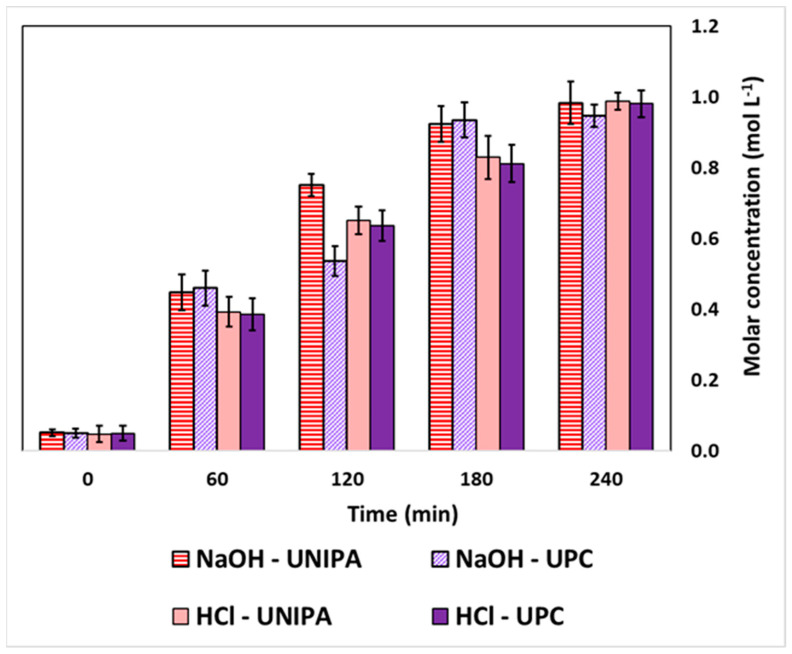
HCl and NaOH concentrations measured at different time intervals for the experiments performed at UNIPA and UPC premises. Initial salt composition: 2 M NaCl. Applied current density: 100 A m^−2^.

Figure 4 presents the HCl and NaOH concentrations measured at different time intervals in the acid and base channels, respectively, obtained with the two laboratory EDBM units at UPC and UNIPA with a starting concentration of 2 M NaCl in the feed compartment and at a current density of 100 A m^−2^.

In both the UPC and UNIPA experiments, the duration of the experiments was 240 min. Since these tests were performed in a closed-loop configuration with constant applied current, the HCl and NaOH concentrations increased over time, as expected. The acidic solution reached a concentration of 0.99 M for UNIPA and 0.98 M for UPC at the end of the experiments. Similarly, the concentration of the alkaline solution was 0.98 M for UNIPA and 0.95 M for UPC at the end of the experiments. Notably, the rate of concentration increase was not constant throughout the test but decreased over time. Indeed, as shown in the first hour of the experiments, the concentration of both the acid and alkaline solutions changed from 0.05 M to 0.45 M for NaOH and from 0.05 M to 0.39 M for HCl. In the last hour of the experiment, the rate of concentration increase fell from 0.40 M h^−1^ to 0.06 M h^−1^ during the experiment for NaOH, whereas it decreased from 0.34 M h^−1^ to 0.16 M h^−1^ during the experiment for HCl. Indeed, the higher the HCl and NaOH concentrations (Figure 4) across the membranes, the larger the effect of non-ideal phenomena such as diffusion and water transport. Moreover, water transport is strongly affected by the osmotic pressure difference across the membranes, which depends on the solution composition and concentrations. Furthermore, the pH of the saline solution decreased with time and stabilised around 2. This can be attributed to the higher mobility of protons compared to hydroxide ions, thus leading to greater acid diffusion than base diffusion into the saline compartment. The concentration trends (in Figure 4) for both acid and base showed small deviations between the results of both institutions. Additionally, the ANOVA analysis was performed for both the acid and alkaline solutions. The analysis of the acid solution determined values of Fc lower than Fcr (0.01 < 4.965). Likewise, for the base solution, the values of Fc were also lower than Fcr (0.033 < 4.965). Therefore, statistically, the effect of performing EDBM experiments at different laboratories on the profile concentrations was not significant.

**Figure 5:**
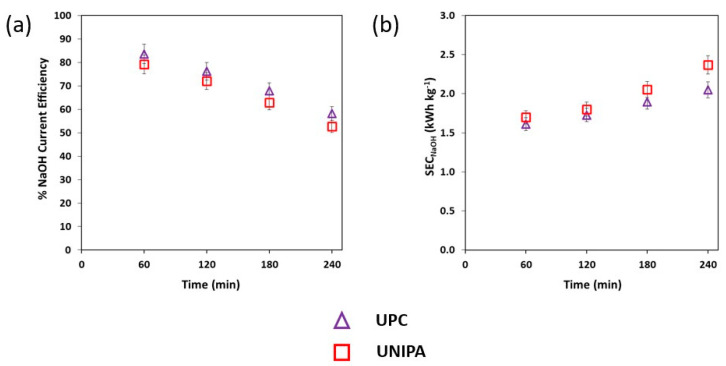
(**a**) Current efficiency and (**b**) specific energy consumption as functions of time for the experiments performed at UNIPA and UPC premises. Initial salt composition: 2 M NaCl. Applied current density: 100 A m^−2^.

Figure 5 shows the SEC and the CE as functions of time for the results obtained at both institutions.

The SEC_NaOH_ increased gradually in the experiments from 1.69 kWh kg^−1^ to 2.34 kWh kg^−1^ at UNIPA and from 1.61 kWh kg^−1^ to 2.05 kWh kg^−1^ at UPC during the experiments (Figure 5b). The ANOVA analysis was also performed with the SEC values to demonstrate reproducibility in the experiments at UNIPA and UPC. The analysis revealed that Fc was lower than Fcr (i.e., 0.892 < 5.318), suggesting that the effect of performing EDBM experiments at different laboratories on SEC values was not statistically significant. Conversely, Figure 5a depicts the CE evolution as a function of time. UNIPA reported a decrease in CE from 79.2% to 52.7%, while UPC reported a reduction in CE from 83.6% to 58.3%. The drop in CE can be attributed to unfavourable phenomena such as back diffusion and water transport across the membranes [33]. The ANOVA analysis also used the CE data to demonstrate the reproducibility at both laboratories. The results showed that Fc was lower than Fcr (i.e., 0.641 < 5.318). As a result, the effect of conducting EDBM experiments at different laboratories was also not statistically significant in terms of CE values.

It can be concluded that considering the ANOVA analysis carried out among the monitored parameters (i.e., HCl and NaOH concentrations, SEC, and CE) from both laboratories, the experimentally collected data were not statistically different. Therefore, the results coming from the two laboratories are reproducible. Once reproducibility was confirmed, the experimental campaign was split between the two institutions to collect a broader range of data.

The results of the analysis at various applied current densities and combinations of membranes are presented and discussed in the following sections.

### 3.2. Effect of Current Density in the Different Types of Membrane Configurations Used in the EDBM Set-Ups

The applied current is one of the most critical parameters of the EDBM operation [34]. Three different values of current densities (i.e., 100, 200, and 300 A m^−2^) were adopted and tested for the two combinations of membranes (i.e., Type A and Type B). In the experiments employed, the initial operating conditions are reported in Table 3. The effects of current density on the EDBM performance were demonstrated in terms of concentrations, SEC, and CE.

The CE is affected by both the applied current and the studied membranes.

**Figure 6:**
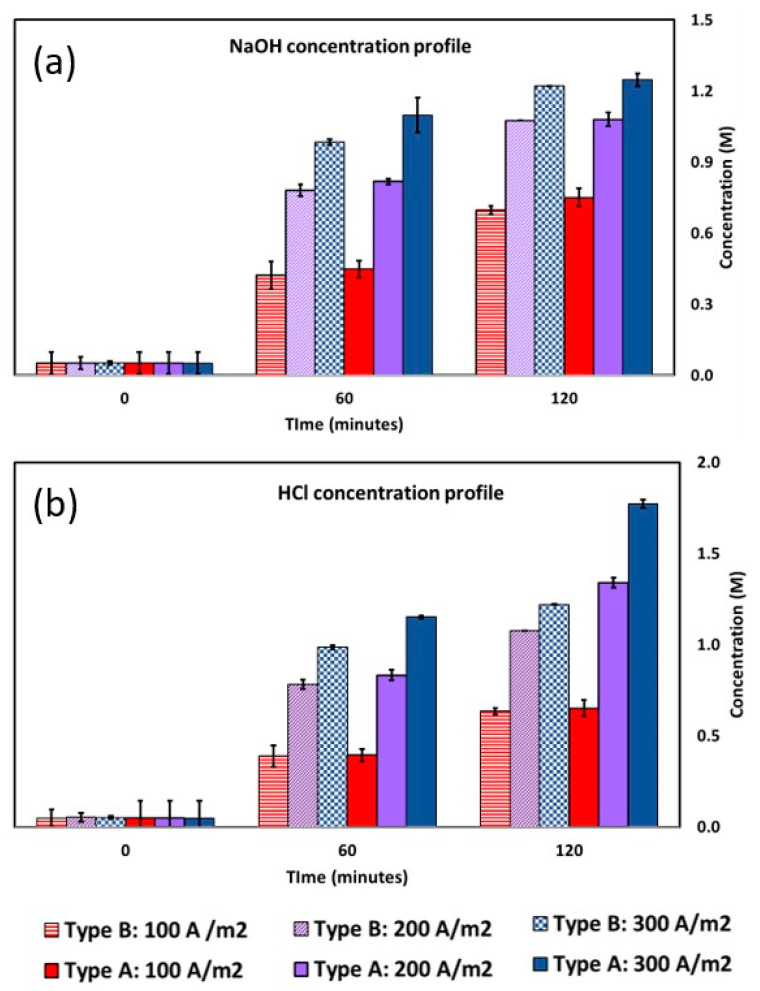
Concentration profile (M) of (**a**) NaOH and (**b**) HCl versus time at three different current densities for Type A and Type B membranes: densities: 300 A m^−2^, 200 A m^−2^, and 100 A m^−2^. Initial NaCl concentration 2 M.

Figure 6 illustrates an upward trend in the concentration values of both Type A and Type B products as the applied current was increased.

**Figure 7:**
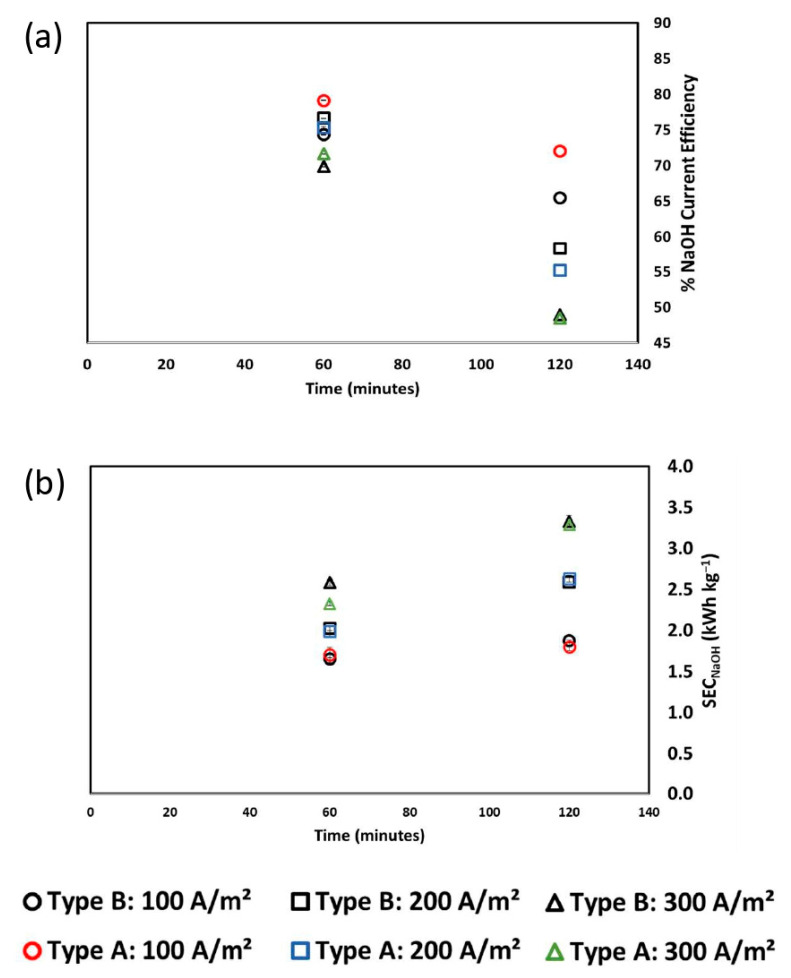
(**a**) Current efficiency and (**b**) specific energy consumption (SEC) versus time at three different current densities: 100 A m^−2^, 200 A m^−2^, and 300 A m^−2^ for Type A and Type B membranes. Initial NaCl concentration in the salt compartment: 2 M.

At the end of the experiments (i.e., after 120 min of operation), Type A exhibited more concentrated acid and alkaline solutions compared to Type B. The most concentrated solutions were generated when applying 300 A m^−2^. Hence, as the current density increased, the final concentration of products attained during the same process time also increased. This was due to the faster rate of water dissociation in the BPM junctions as the applied current increased. Figure 7a shows the current efficiency profiles of both types of membranes over time when varying the applied current density.

The CE decreased over time regardless of the applied current or the type of membranes, which can be related to two major phenomena. Firstly, there is a higher acid and base ion diffusion into the salt compartments as the process progresses. In fact, since the experiments were conducted in a closed loop, the acid concentration gradient across the AEM and the base concentration gradient across the CEM increased over time. Therefore, an increase in the acid/base diffusion phenomenon to the saline compartment is expected over time. Other phenomena are mainly related to the non-ideal permselectivity of the IEMs and water transport. Moreover, the BPM non-ideal behaviour reduced the production of protons and hydroxide ions, which was partially replaced by the transport of sodium and chloride ions through the BPM layers [35]. At 100, 200, and 300 A m^−2^, the CEs for Type A at the end of the test were 72.0%, 55.2%, and 48.5%, respectively. On the other hand, the CEs for Type B at the end of the test were 65.45%, 58.3%, and 48.91%, respectively. This trend is due to the different acid/base concentrations reached at the end of the tests. In contrast, with the same target concentration, the lower the applied current, the lower the current efficiency. This is due to the greater contribution of the diffusive flux to the total ion flux at low applied currents. Figure 7b depicts the SEC at 100, 200, and 300 A m^−2^. The SEC increased over time, regardless of the applied current. This was the result of two opposing effects. On the one hand, the applied voltage is reduced over time, thus reducing the SEC. On the other hand, as previously observed; there was a decrease in CE. The latter phenomenon prevailed, resulting in an overall increase in the SEC. When operating at 100, 200, and 300 A m^−2^, the final SEC values for Type A were 1.80, 2.63, and 3.29 kWh kg^−1^, respectively. Similarly, Type B reported SEC values of 1.87, 2.9, and 3.33 kWh kg^−1^, respectively.

Results were compared with the previously published literature (see Table 4) [19,20,21,22]. In the present work, the CE (%) values were lower compared to the previous studies reaching a maximum of 72% with Type A with an average SEC of about 2 kWh kg^−1^_NaOH_. The average CE values obtained, as shown in Table 4, are consistent with those reported in the literature. Reig et al. [20] obtained the highest performance indicators, likely due to a lower effect of non-ideal phenomena (e.g., non-perfect stack sealing or misalignment of the membranes and spacers, or even micro-holes in the membranes). In addition, the SEC values obtained in the present work are among the lowest recorded in the literature due to the operating conditions utilised. Indeed, in the present work, the applied maximum current density was 300 A·m^−2^, whereas most of the previous results used a higher current density (see Table 4). This study showed that the higher the acid and base concentrations achieved over time, the lower the CE and, consequently, the higher the SEC. For example, when high acid and base concentrations are obtained, the higher diffusive flows across the monopolar membranes to the saline compartment result in a significant reduction in current efficiency. Additionally, the high pH gradient across the bipolar membrane causes an increase in the Nernst potential. The two latter phenomena lead to a rise in SEC. The operating time reported in the literature varies greatly. However, the operating time is proportional to the volume of the solutions in the external tanks. At the same operating and design conditions, a longer time will be required as the volume of solutions increases.

Furthermore, when comparing the five different types of membranes shown in Table 4, the SUEZ membrane produced acid and alkaline solutions with similar concentrations to those obtained with PCCell membranes. SUEZ membranes had a relatively low SEC and one of the largest active membrane areas studied. Further research could focus on technical–economic analyses to investigate the profitability of EDBM technology using various types of membranes.

### 3.3. Performance Parameters of the Two Types of Membranes

This section compares the performance of the two combinations of membranes (i.e., A and B) investigated in this study. The comparison was carried out in four different scenarios, each corresponding to two different NaOH concentrations reached, either 0.4 M or 0.8 M, and two different applied current densities, either 100 or 300 A m^−2^, corresponding to the lower and upper values of the current density range investigated in this work. These scenarios are reported in Table 5.

**Figure 8:**
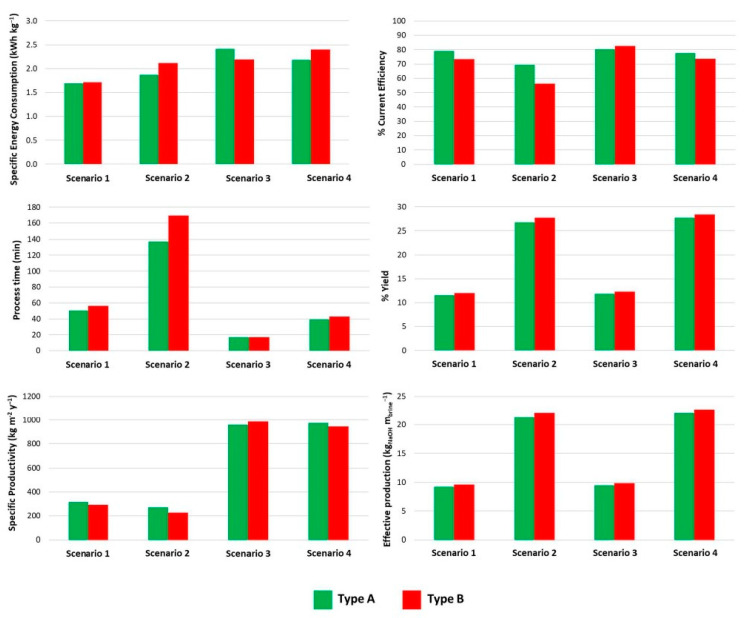
Comparison between the two types of membranes A and B in terms of specific energy consumption, % Current efficiency, process time, yield, specific productivity, and effective production for each investigated scenario.

Histograms were used to compare the following performance parameters: SEC, CE, Yield, SP, Effective production, and Production time. The performance parameters were determined based on NaOH since it is the most valuable commercial product. The histograms of the performance parameters are reported in Figure 8.

Type A is distinguished from Type B by its ultra-thin non-woven structure, as previously stated. This feature provides Type A with a proton blocker property, resulting in more concentrated acid and alkaline streams. The EDBM analysis, however, should not be limited to the expected final concentration of the acid and base streams; performance indicators should also be considered.

Examining the results in Figure 8, it is clear that the two types of membranes behaved differently depending on the applied current (i.e., 100 or 300 A m^−2^) and target NaOH concentration (i.e., 0.8 or 0.4 M). Performance parameters changed when varying the applied current. When operating at a lower electric current (i.e., at 100 A m^−2^ in this case), the SEC values are also reduced. The two types of membranes behaved similarly at 0.4 M NaOH and 100 A m^−2^; yet, at higher electric currents, the SEC values varied significantly from one type of membrane to the other. An increase in the electric current can lead to possible voltage drops that are related to a rise in the SEC values and an increase in non-ideal phenomena (e.g., diffusion of the ion species and water transport across the membranes). The increase in SEC is primarily due to a lower CE caused by non-ideal phenomena. Overall, it can be noted a higher CE of Type A membranes was offset by the higher stack voltage, resulting in a very similar SEC as previously mentioned.

However, the performance varied in favour of Type A membranes at a target of 0.8 M NaOH, as better SEC, CE, SP, and process times were obtained. Specifically, compared to scenario 1, process times were 2.7 and 3 times longer for membranes A and B, respectively. The required process time was not exactly double as expected theoretically (assuming perfect perm-selective membranes and the absence of non-ideal phenomena). Indeed, this resulted from non-ideal phenomena occurring during the EDBM process, especially water transport (i.e., osmosis and electro-osmosis), which tends to dilute the alkaline solution. Furthermore, the applied electric potential decreased during the process due to the reduction of the ohmic resistance of the electrolyte solutions. In contrast, the Nernst potential increased as the concentration of acid and base increased. Overall, the reduction in ohmic resistance prevailed, thus determining a downward trend of the EDBM electric potential during the process. Therefore, since in this study the experiments were carried out in a closed-loop configuration, the higher the target concentration, the lower the resulting average electric potential. Finally, the effective production was 21.4 kg_NaOH_ m_brine_^−3^ and 22.2 kg_NaOH_ m_brine_^−3^, respectively and thus, more than doubled compared to the values obtained at the target of 0.4 M.

When operating at 300 A m^−2^ and targeting 0.4 M NaOH, the best-performing membranes were Type B because all indicators (except for the processing time) were better than those obtained for Type A. The increase in current density to 300 A m^−2^ had a clear impact on the process time since the acid/base production rate (at least theoretically) was increased [36]. Moreover, as expected, the SEC was higher when operating at 300 A m^−2^. Indeed, the SEC is related to the CE and the electric potential, as mentioned before. The electric potential is partly related to the applied current according to the first Ohm’s law (Ohmic contribution) and partly to the Nernst potential. The results showed a predominant effect of the Ohmic contribution, thus increasing the electric potential with a consequent rise in SEC to 2.4 kWh kg^−1^ for membranes A and to 2.2 kWh kg^−1^ for membranes B.

Finally, at 300 A m^−2^ and a target NaOH concentration of 0.8 M, the best-performing membranes were Type A, as SEC, process time, CE, and SP were higher than those of Type B membranes. In comparison to scenario 2, the processing time in scenario 4 was reduced not only because of the higher current density but also because of the higher CE. Indeed, the CEs in scenario 4 were 9% and 17% higher than those obtained in scenario 2 for Types A and B, respectively. Unlike scenario 3, there was no clear trend for the SEC, resulting in 2.28 kWh kg^−1^ for Type A and 2.4 kWh kg^−1^ for Type B. The SEC values were more affected by CE and less by electrical potential. Indeed, regardless of the membrane type used, when targeting 0.8 M, the electric potential decreased by less than 5% compared to that found in scenario 3 at 0.4 M NaOH. On the other hand, the CE varied by only 3% for membranes A and 11% for membrane B when passing from 0.4 M (i.e., scenario 3) to 0.8 M (i.e., scenario 4) of the target, thus indicating a slightly better selectivity of Type A than B. In comparison to the corresponding scenario at 100 A m^−2^ (i.e., scenario 2), the yield (27.7–28.4%), SP (974–943 kg m^−2^ y^−1^), and effective production (22.2–22.7 kg_NaOH_ m_brine_^−1^) did not differ significantly for Types A and B, hence demonstrating that diffusion and water transport phenomena did not have a significant influence on performance. Additionally, the ANOVA analysis was performed to discuss the statistical variance between Type A and Type B. The results from the statistical analysis are detailed in Table 6. Specifically, Table 6 shows the values of Fc obtained by considering SEC, CE, yield, SP, effective production, and production time for both Type A and Type B.

All the values of Fc shown in Table 6 are below the Fcr. Therefore, the performance parameters obtained using the two types of membranes were not statically different among them. This fact confirmed that using either Type A or Type B did not significantly influence the behaviour of the EDBM.

## 4. Conclusions

The EDBM experimental interlaboratory campaign devoted to generating HCl and NaOH from brines using new CEMs and AEMs (Suez) incorporating thin and ultrathin non-woven polyester and polypropylene supports provided a high reproducibility as demonstrated by the ANOVA analysis.

A direct effect of current density was observed on the HCl and NaOH final concentrations. For instance, by working at concentrations of 2 M NaCl, regardless of the type of membranes used, it was possible to reach NaOH concentrations higher than 1 M at 300 A m^−2^. Similarly, under the same conditions, HCl concentrations were higher than 1 M, reaching a maximum concentration of 1.7 M.

A decrease in current efficiency over time was observed. For example, after 120 min, the current efficiency for membranes incorporating thin-polypropylene (Type A) decreased to 72%, 55%, and 48% working at 100, 200, and 300 A m^−2^, respectively. This effect can be caused by: (i) higher acid and base diffusion towards the salt compartment and (ii) the non-ideal permselectivity of the IEMs. Although the ultra-thin structure of the AEM (AR118U) was claimed to have improved properties as a proton-blocking membrane allowing for higher concentrations in acid and alkaline streams (e.g., up to 1 M), there was no statistically significant acid and base concentration difference between Types A and B. Contrastingly, regarding SEC, Type A reported lower SEC values than Type B. However, the values were demonstrated to not be statistically different between Types A and B. Finally, regarding SP, Type A showed higher values than Type B.

## Figures and Tables

**Table 1:** The main characteristics of some membranes used for EDBM.

Manufacture	Membrane	Identification	Functional Groups	Ion-Exchange Capacity	Wet Thickness (μm)	Membrane Support	Permselectivity (%)	Areal Resistance (ohm cm^2^)	Ref.
^1^ SUEZ	AEM	AR103P	quaternary ammonium	2.37 (meq/dry g resin)	570	Woven polypropylene	92	^a^ 9.4	[30]
	CEM	CR61P	sulfonic acid	2.2 (meq/dry g resin)	580		94	^a^ 10	
	AEM-treated	^4^ AR103A tr.	quaternary ammonium	2.37 (meq/dry g resin)	300		92	n.a.	
^1^ SUEZ	AEM	^2^ AR103N	quaternary ammonium	2.37 (meq/dry g resin)	300	Thin non-woven polypropylene	92	^a^ 2.8	[30]
	AEM	^3^ AR118U	quaternary ammonium	2.37 (meq/dry g resin)	130	Ultra-thin non-woven polyester cloth	90	^a^ 11	
	CEM	^2,3^ CR61N	sulfonic acid	2.2 (meq/dry g resin)	300	Thin non-woven polyester cloth	95	^a^ 3.6	
	AEM-treated	^2,3,4^ AR103N tr.	quaternary ammonium	2.37 (meq/dry g resin)	300		92	n.a.	
LLC Innovative Enterprise, Schekinozoat	AEM	MB-1	quaternary ammonium	4.0 (eq/L)	<900	Polystyrene divinylbenzene with polyethylene binder	98	n.d.	[31]
	CEM		sulfonic acid	1.4 (eq/L)	<900		98	n.d.	
Membranes International, Inc.	AEM	BMI-9000	quaternary ammonium	1.3 (meq/dry g resin)	450	Gel polystyrene crosslinked with divinylbenzene	91	^b^ <40	[28]
	CEM		sulfonic acid	1.6 (meq/dry g resin)	450		91	^b^ <30	

^1^ SUEZ BPMs are not cited in this table since they are the combinations of treated AEMs with CEMs. ^2^ Type A membranes used for the experimental campaigns in the present work. ^3^ Type B membranes used for the experimental campaigns in the present work. ^4^ AEMs with catalyst (i.e., immobilised iron II) by immersing them in transition metal solution. ^a^ Areal membrane resistance measured in 0.1N NaCl (meq/dry). ^b^ Areal membrane resistance measured in 0.5 M NaCl (meq/dry).

**Table 2:** Differences between UPC and UNIPA EDBM lab scale set-ups.

	UPC	UNIPA
Pumping system	4 centrifugal pumps (BED 1–4 from PCCell GmbH)	4 peristaltic pumps (BT601S from Lead Fluid Technology, Co., Ltd., Baoding, China)
Sampling	Samples are taken from tanks coupled with ball valve taps	Samples are taken from the tanks using syringes.
Power Supply	HCS-3202	BK precision 1902B
Volume measurement	Graduated vessels allow for monitoring volume variations	The volume is calculated as a function of mass and density. The mass variation is monitored using an analytical scale.

**Table 3:** Operational conditions adopted in EDBM experiments, including current density, initial concentrations of the different streams, flow rates, and initial volumes of the product containers.

Current Density	Channel	Initial Concentration	Initial Volume	Flow-Rate
100–300 A m^−2^	Acid	0.05M HCl	1.0 L	50 L h^−1^ *
	Base	0.05M NaOH	1.0 L	50 L h^−1^ *
	Salt	2M NaCl	1.5 L	50 L h^−1^ *
	ERS	0.25M Na_2_SO_4_	1.5 L	50 L h^−1^ *

* Corresponding to a mean channel flow velocity of 7 cm s^−1^.

**Table 4:** Comparison between the experimental data collected in the present work and others published in the literature.

Set-Up			Experimental Conditions					Concentrations (mol/L)			Performance Parameters		Ref.
Membranes		Membrane Stack	Active Area (cm^2^)	Nº Triplets	Current Density (A·m^−2^)	Operation Time (min)	Operation Mode	Initial Salt Solution	Final HCl	Final NaOH	SEC (kWh/kg)	CE (%)	
SUEZ	AEM: AR118U & AR103N	HDWR (MK-1) Suez	280	5	100–300	120	Batch	2 M NaCl	1.77	1.25	3.29	48.5	This work
	CEM: CR61N												
	BM: AR103N-treated layered with CR61N												
P CCell	CEM: PC-SK	PCCell ED64-004	64	3	520	405	Batch	3.2 M NaCl	2.01	1.87	2.71 (NaOH)	88–55	[20]
	AEM: PC Acid 60												
	BM: PC BP												
M EGA	CEM: M-PP RALEX	Elektrolyse Project	100	1	1000	2400	Semi-batch	1 M NaCl	3.17	3.63	41.0 (HCl)	n.d.	[21]
	AEM: M-PP RALEX												
Fumatech	BM: Fumasep FBM												
Qianqiu	CEM	Shandong Tianwei Membrane Technology Co., Ltd.	88	3	570	140	Batch	Pre-treated RO concentrate	0.65	n.d.	9.0 (HCl)	n.d.	[19]
	AEM												
Fumatech	BM												
Neosepta	CEM: Neosepta CMX	PC-Cell EDQ380	385	8	121	-	Single-pass	0.19 M NaCl	0.15	0.14	3.65 (HCl)	80	[22]
	AEM: Neosepta ACM												
	BM: Neosepta BP-1												

**Table 5:** List of investigated scenarios.

	Current Density (A m^−2^)	NaOH Target Concentration
Scenario 1	100	0.4 M
Scenario 2	100	0.8 M
Scenario 3	300	0.4 M
Scenario 4	300	0.8 M

**Table 6:** ANOVA test performed for the performance parameters of Type A and Type B membranes.

Critical F_value_ (Fcr) = 5.9874	
	F_calculated_ (Fc)
SEC	0.0849
CE	0.7251
Yield	0.0098
Process Time	0.0604
SP	0.0037
Efficiency	0.0098

## Data Availability

The data used to support the findings of this study are available on the open-source repository—Zenodo.

## Nomenclature

**Acronyms/Abbreviations**	
AEM	anion exchange membrane
ANOVA	ANalysis of VAriance
BMED	bipolar membrane electrodialysis
BPM	bipolar membrane
CEM	cation exchange membrane
CRM	critical raw material
EDBM	electrodialysis with bipolar membranes
EI	economic importance
EU	European Union
IEM	ion-exchange membrane
MLD	minimum liquid discharge
SR	supply risk
SWRO	sea water reverse osmosis
UNIPA	UNIversità degli studi di PAlermo
UPC	Universitat Politècnica de Catalunya
ZLD	zero liquid discharge
**Symbols**	
α	significance level parameter
$C_{b,NaOH,0}$	concentration of NaOH at time 0, mol L^−1^
$C_{b,NaOH,t}$	concentration of NaOH at time t, mol L^−1^
*CE*	current efficiency, %
*F*	faraday constant, C mol^−1^
Fc	calculated F-value
Fcr	critical F-value
*I*	applied electric current, A
$M_{NaOH}$	NaOH mole mass, kg. mol^−1^
*N*	number of triplets
*S*	active membrane area, m^2^
*SEC*	specific energy consumption, kWh kg^−1^
*SP*	specific productivity, kg m^−2^ y^−1^
*t*	operation time, s
*U*	EDBM voltage, V
$V_{b,0}$	volume of the base solution at time 0, L
$V_{b,t}$	volume of the base solution at time t, L
$V_{s,t_{0}}$	volume of the salt solution at time 0, L
